# miRNA-375 a Sensor of Glucotoxicity Is Altered in the Serum of Children with Newly Diagnosed Type 1 Diabetes

**DOI:** 10.1155/2016/1869082

**Published:** 2016-05-24

**Authors:** Lucien Marchand, Audrey Jalabert, Emmanuelle Meugnier, Kathleen Van den Hende, Nicole Fabien, Marc Nicolino, Anne-Marie Madec, Charles Thivolet, Sophie Rome

**Affiliations:** ^1^CarMeN Laboratory (INSERM 1060, INRA 1362, INSA), Lyon-Sud Faculty of Medicine, University of Lyon, Chemin du Grand Revoyet, 69600 Oullins, France; ^2^Hospices Civils de Lyon, Lyon-Sud Hospital, Department of Diabetology and Endocrinology, 69495 Pierre-Bénite, France; ^3^Hospices Civils de Lyon, Department of Pediatric Endocrinology, Femme-Mère-Enfant Hospital, 69500 Bron, France; ^4^Hospices Civils de Lyon, INSERM U851, Lyon-Sud Hospital, Department of Immunology, 69495 Pierre-Bénite, France

## Abstract

*Background.* The use of miRNAs as biomarkers for Type 1 Diabetes (T1D) risk is attractive as T1D is usually diagnosed in front of acute symptoms. As miR-375 is highly expressed in the endocrine pancreas, we postulated that its circulating level might reflect beta cell alterations and might be altered in the blood of T1D patients recently diagnosed.* Methods.* Sera were obtained from 22 T1D children at onset of the disease, before subcutaneous insulin treatment, and from 10 nondiabetic pediatric controls. MiR-375 seric level was quantified by stem-loop RT-PCR-based assay. MiRNAs regulations in isolated human islets in response to high glucose concentrations were determined by TaqMan Low-Density Array.* Results.* The abundance of miR-375, among the 410 miRNAs detected in human islets, mirrored its well-established role in rodent islet biology. Upregulated miRNAs targeted genes involved in islet homeostasis and regulation of beta cell mass. Downregulated miRNAs, including miR-375, were involved in pancreas secretion and protein turnover. Seric level of miR-375 was lower in T1D children versus age-matched controls, without any correlations with HbA1c, glycaemia, and number of autoantibodies.* Conclusion.* Altered circulating level of miR-375 at onset of T1D might be a general biomarker of metabolic alterations and inflammation associated with the disease.

## 1. Introduction

Type 1 diabetes (T1D) affects approximately 10% of adults diagnosed with diabetes [1]. First described as an autoimmune disease in which autoreactive T cells destroy insulin-producing beta cells of the pancreas, it is now recognized that several potential environmental factors, including the possible role of chronic viral infections, can be causative agents [2]. Although it is important to diagnose diabetes as early as possible in order to prevent ketoacidosis, circulating autoantibodies against beta cell autoantigens are currently the only biomarkers available in the clinics. However, the presence of these autoantibodies does not always correlate with the loss of beta cell mass as positive individuals do not always develop T1D [3]. In addition, recent access to pancreatic tissue from the nPOD biobank has allowed showing the existence of a significant amount of remaining beta cells in the pancreas of T1D patients several years after clinical diagnosis [4, 5]. Therefore, identification of new biomarkers in the early phases of the disease is necessary and represents an important challenge, as the disease is largely silent in its initial stages.

In that context, blood miRNA levels represent a new class of biomarkers for diagnosis and prognosis of several diseases and have emerged as new targets for treatments and interventions. MiRNAs are small noncoding RNAs of 19–22 nucleotides which act as negative regulators of gene expression, mainly at the posttranscriptional level [6]. Studies in the last 5 years have demonstrated that miRNAs are not only found intracellularly but are also detected outside cells in various body fluids [7]. Remarkably, it has been found that some circulating blood miRNA levels are proportional to the degree of severity of the pathology such as drug-induced liver injury [8], cardiovascular infection [9], cancer [10], Alzheimer's [11], inflammation [12], and metabolic diseases (obesity and type 2 diabetes [13]). In the context of T1D, 12 miRNAs were found more concentrated in sera from children and adolescents with newly diagnosed T1D compared to sera from age-matched controls [14]. Among them, an association of miR-25 with improved glycemic control and better residual beta cell function was found, suggesting that this miRNA could be used during early and intensive management of newly diagnosed diabetes to improve blood glucose control and reduce microvascular complications [14]. Another recent study involving pediatric T1D patients with duration of disease longer than 1 year revealed a significant deregulation of miR-21, miR-126, and miR-210 in plasma and urinary samples. As dysregulation of these miRNAs has been shown in type 2 diabetes or renal diseases, their alterations in plasma of T1D patients may indicate ongoing endothelial dysfunction or preclinical kidney disease [15]. Taken together, these 2 studies provided a proof of concept that the concentration of circulating miRNAs was affected in both newly or long-term diagnosed T1D already under insulin treatment. However, no data indicate whether these blood miRNAs could be monitoring the course of the disease in the early steps.

MiR-375 is highly expressed in the endocrine pancreas (islet beta cells and nonbeta cells) and was first cloned from an insulin-secreting cell line MIN6B1 [16]. In vitro studies have shown that miR-375 is involved in islet development [17], inhibits insulin gene expression in response to glucose [16], and regulates voltage-gated Na(+) channels and the exocytotic machinery [18].* KO-miR-375 *mice are hyperglycemic and have elevated plasma glucagon levels [17]. Based on these data, we hypothesized that the level of miR-375 could be altered in the blood of T1D patients of recent onset. Thus, in this study, we have validated this hypothesis by quantifying the level of miR-375 in the sera from children at onset of T1D (before subcutaneous insulin treatment) and in age-matched controls. We have also analyzed the global miRNA profile in human islets exposed to high glucose concentrations to determine whether glucotoxicity might affect the level of miR-375 in beta cells.

## 2. Methods

### 2.1. Patients

Sera from 22 children at onset of T1D within two days after clinical diagnosis (immediate insulin-requiring diabetes with at least one positive autoantibody) and before subcutaneous insulin treatment and sera from 10 control subjects (nonobese and nondiabetic child) were collected in 2014 at the Pediatric Endocrinology Department (Hospital Femme-Mère-Enfant, Bron, France). Exclusion criteria for both groups were any febrile illness during the last month and chronic inflammatory disease. Age, gender, BMI, glycemia at discovery, and HbA1c were recorded. Blood samples were taken during usual biological monitoring. All patients and controls provided written informed consent for the collection of samples and subsequent analysis. Clinical and biological characteristics of T1D children and controls are summarized in Table 1.

### 2.2. Serum Sampling

Whole blood was collected and allowed to clot for 30 min at room temperature. Clot was removed by centrifuging at 2,000 ×g for 10 minutes in a refrigerated centrifuge. The resulting supernatant designated as serum was transferred into a clean polypropylene tube using a Pasteur pipette. Autoantibodies to glutamic acid decarboxylase (GAD), islet-antigen 2 (IA2), and zinc transporter 8 (ZnT8) were quantified by ELISA (Medizym®, Medipan GmbH, Dahlewitz, Germany) and were considered to be positive for titers above 5, 10, and 15 UI/mL, respectively.

### 2.3. Serum Hemolysis Quantification

Hemolysis of sera (80 *μ*L) was evaluated by spectrophotometry (450 nm) (Biochemical Department, Lyon-Sud Hospital). None of the 32 serum samples were hemolysed.

### 2.4. Culture of Human Islets

Human islets were obtained in collaboration with the Geneva University Hospitals Cell Isolation And Transplantation Center (Switzerland) and from the Unité Mixte de Thérapie Cellulaire et Tissulaire de Grenoble (France) Characteristics of the donors are summarized in Table 2. The use of human islet preparations for experimental research was approved by the Institutional Review Board for Clinical Research of the Departments of Neurology, Dermatology, Anesthesiology and Surgery of the University Hospital of Geneva (CER number 05–028). Islets (purity at least 70%) were processed as described previously [19] and cultured in a DMEM medium containing 5.5 mM or 16.7 mM glucose and 5% (vol./vol.) FBS (PAA Laboratories GmbH).

### 2.5. RNA Extraction from Human Serum

 150 *μ*L of serum was transferred in an Eppendorf tube and mixed with 450 *μ*L of TRIzol® LS specially designed for liquid samples (Life Technologies). Then, 150 *μ*L of chloroform (Sigma Aldrich) was added and 20 fmol of synthetic cel-miR-39 (UCACCGGGUGUAAAUCAGCUUG) was spiked into the serum (Qiagen). Cel-miR-39 from* C. elegans* has no homology with human miRNAs. It is added at the beginning of the extraction as control for RNA extraction. The solution was vortexed to obtain a homogenous phase. Tubes were centrifuged for 20 minutes at 12,000 rpm (4°C). A fixed volume of aqueous phase (250 *μ*L) was mixed with 250 *μ*L isopropanol and 2 *μ*L glycogen (Roche Diagnostic) and tubes were centrifugated for 30 minutes at 13,000 rpm. The pellet was washed with ethanol 75% and dried and the pellet resuspended in 16 *μ*L RNAse-free water.

### 2.6. Amplification of Seric miR-375 by qRT-PCR

A fixed volume of total RNA (5 *μ*L for miR-375 and 1 *μ*L for miR-39) was used for reverse transcription (RT) reaction. RT reactions were realized with 0.15 *μ*L dNTP, 1.5 *μ*L buffer (10x), 9 *μ*L RNAse-free water, 0.2 *μ*L RNAse inhibitor, 1 *μ*L multiscribe RTase, and 3 *μ*L of specific miR-375 RT primers (Life Technologies). RT reactions were realized at 16°C for 30 minutes, 42°C for 30 minutes, and 85°C for 5 minutes using a PTC-100 Peltier thermal cycler (Mj Research). At the end of the RT reaction, samples were diluted to 1/10 with RNAse-free water for PCR.

The TaqMan miR-375 assay (Life Technologies) was used to quantify miR-375. 5 *μ*L of RT reactions was mixed with 10 *μ*L of TaqMan Universal PCR master mix (×2, (Life Technologies)), 4 *μ*L of RNAse-free water, and 1 *μ*L of TaqMan miRNA Assay (×20, (Life Technologies)). PCR was performed on a Rotor Gene thermocycler at 95°C for 10 minutes, followed by 40 cycles of 95°C for 15 seconds and 60°C for 60 seconds. All samples were run in duplicate; Ct (cycle threshold) values were automatically determined by the software.

### 2.7. Statistical Analysis

Analyses were performed with R software. *t*-tests were used for quantitative variable. Pearson's correlations between miRNA levels and clinical or metabolic parameters were calculated.

### 2.8. miRNA Profiling by qRT- PCR in Human Islets

Total RNA was extracted from 3 independent human islet preparations by using TriPure Isolation Reagent (Roche Applied Science, France). RNA concentration was measured with a NanoDrop spectrophotometer, ND-1000 (Thermo-Fisher, Waltham, MA, USA). Profiling of miRNAs was determined using the TaqMan Low-Density Array cards A and B (Applied Biosystems). Briefly, total RNA was reverse transcribed using TaqMan miRNA Reverse Transcription Kit (Applied Biosystems) in combination with the stem-loop Megaplex primers in 7.5 *μ*L. Next, 2.5 *μ*L of RT was mixed with 2.5 *μ*L of Megaplex PreAmp Primers and 12.5 *μ*L TaqMan PreAmp Master Mix in a 25 *μ*L PCR reaction and preamplified. The preamplified cDNA was then diluted with 0.1x TE (pH 8.0) to 100 *μ*L. Nine *μ*L of diluted cDNA solution was used for qRT-PCR runs. qRT-PCR was performed on a 7900HT thermocycler (Applied Biosystems) using the manufacturer's recommended cycling conditions: 50°C for 2 min and 95°C for 10 min and then 40 cycles at 95°C for 15 s and 60°C for 1 min. Data were recorded at the end of each cycle. Cycle threshold (Ct) values were calculated with the SDS software using automatic baseline settings with assigned minimum Ct threshold of 0.2. Ct values > 40 were excluded from data analysis. Each card included 3 miRNA endogenous controls and one miRNA assay not related to human. For each card, quality controls were performed on the raw data by checking internal controls and using box plot diagrams. Since the currently used normalization small RNA mammU6 plotted in each card was not stably expressed in our different samples, we have used the mean expression level of all fully observed miRNAs for normalization [20]. Group comparisons were made by using student's *t*-test (*p* < 0.05) on normalized data, to select the differentially expressed miRNAs.

## 3. Results

**Figure 1:**
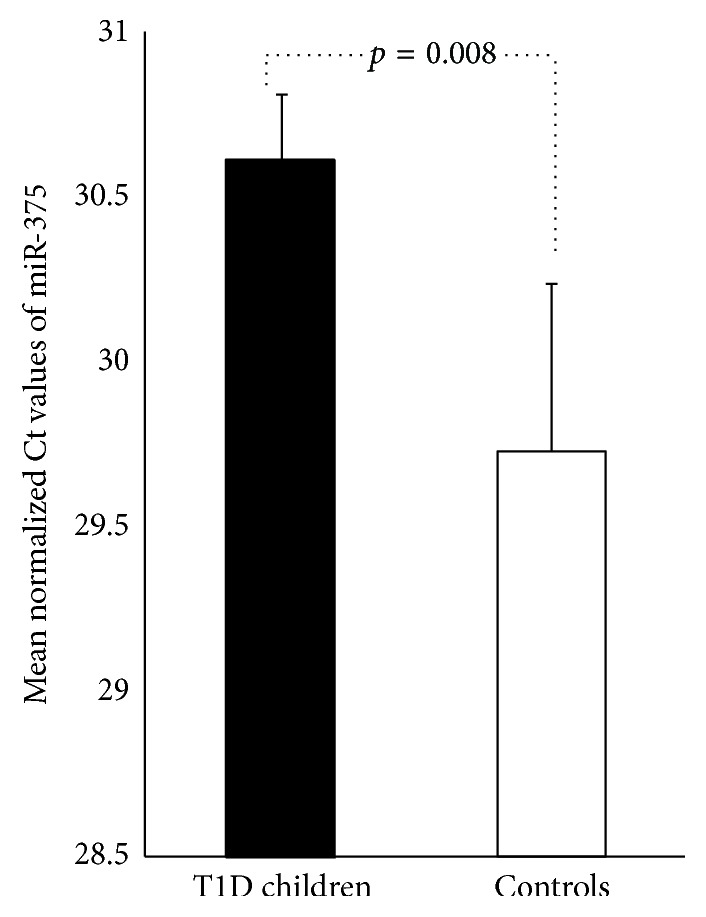
Quantification of miR-375 in sera of newly diagnosed T1D children. Data are expressed as Ct values. The Ct is defined as the number of cycles required for the fluorescent signal to cross the threshold (i.e. exceeding background level). Ct levels are inversely proportional to the amount of target nucleic acid in the sample (i.e. the lower the Ct level, the greater the amount of target nucleic acid in the sample). To analyze individual qPCR data, we first controlled the quality of RNA extraction by quantifying Cel-miR-39 (spike). The following formula was used for normalizing the Ct values of miR-375 in all sera samples: raw Ct value − [(spike-in average Ct value of sample) − (median spike-in Ct)]. Data are expressed as mean values ± SEM.

This study aimed to evaluate whether the circulating level of miR-375 was altered at onset of T1D. Therefore, we have quantified miR-375 in sera from children population at onset of T1D (immediate insulin-requiring diabetes with at least one positive autoantibody) before subcutaneous insulin treatment. T1D children had elevated HbA1c (11.82% ± 2.15) and had at least one positive autoantibody for GAD, IA-2, and/or ZNT8, confirming an ongoing autoimmunity to beta cells. As shown in Figure 1, the level of miR-375 was significantly lower in the sera of the 22 T1D children than in the sera of the 10 healthy controls (mean normalized Ct values: 30.61 ± 0.20 versus 29.02 ± 0.57, *p* = 0.008). We did not find any association between miR-375 serum concentration and HbA1c, glycaemia, and the number of autoantibodies (Table 3).

**Figure 2:**
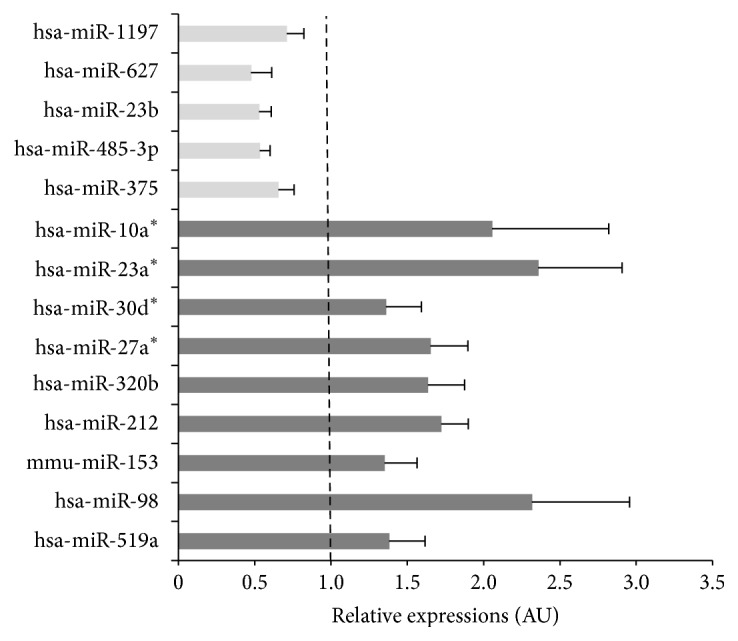
MiRNAs are regulated in human islet in response to glucose. miRNA regulations in human islets exposed to 16.7 mM versus 5.5 mM for 24 h (see Table 2). Data are expressed as mean of 3 biological replicates ± SEM. Each miRNA was normalized to the geometric mean of all miRNAs expressed in all replicates.

Then, we have analyzed the global miRNA profile in human islets exposed to high glucose concentrations to determine whether glucotoxicity might affect the level of miR-375 in beta cells. As shown in supplementary Table 1 in Supplementary Material available online at http://dx.doi.org/10.1155/2016/1869082, 410 miRNAs were detected in human islets and miR-375 was the most expressed one. Nine miRNAs (miR-519a, miR-212, miR-320b, miR-27a^*∗*^, miR-30d^*∗*^, miR-23a^*∗*^, miR-30d^*∗*^, miR-23a^*∗*^, and miR-10a^*∗*^) and 5 miRNAs (miR-375, miR-485-3p, miR-23b, miR-485-3p, miR-23b, miR-627, and miR-1197) were up- or downregulated by glucose, respectively (Figure 2). miR-375 and miR-212 had been previously identified as regulated by glucose in rat islets [21]. Upregulated miRNAs targeted important signaling pathways for islet homeostasis and regulation of beta cell mass. They were also involved in viral infections and cancer development (see Box 1).

Downregulated miRNAs were involved in pancreas secretion and in protein processing and degradation (see Box 1). All these functions are important for insulin secretion. In addition, downregulated miRNAs targeted genes involved in biotin metabolism and glycosphingolipid biosynthesis, two functions affected in T1D patients [22, 23]. Interestingly, miR-375 was downregulated in human islets exposed to high glucose concentrations, suggesting a close relationship between miR-375 and glucose (Figure 2).

## 4. Discussion

The contribution of miRNAs to the development of human T1D has been largely unexplored. More particularly, their use as biomarkers to monitor the fate and/or function of the pancreatic beta cells is attractive. In this study, we have found that miR-375 is strongly decreased in the serum of newly identified T1D children, before the initiation of subcutaneous insulin treatment. These data are in agreement with previous studies on animal models showing that plasma miR-375 is altered in streptozotocin-treated mice prior to the onset of hyperglycemia or 2 weeks before diabetes onset in nonobese diabetic mice [24]. As miR-375 regulates insulin secretion and beta cell mass [16, 17], we postulated that its expression might be altered in beta cells concomitantly to its circulating level. In order to further interpret our data, we have determined, for the first time, the miRNA profile of human islets incubated with high glucose concentrations. The abundance of miR-375 in the miRNA profile of human islets found in this study confirmed its critical role in human pancreatic beta cells [25], mirroring its well-established role in rodent islet biology [16]. We found that miR-375 was downregulated in human islets treated with high glucose concentrations. Decreased functional beta cell mass is a hallmark of both type 1 diabetes and type 2 diabetes (T2DM) and, interestingly, obese and pre-T2DM patients have also decreased level of blood miR-375 compared to healthy controls [26–28]. On the contrary, T1D and T2DM patients with controlled glycemia have increased circulating levels of miR-375 [29–31]. As we have previously found that beta cells can release miR-375 in extracellular environment [32], our data could suggest a possible link between circulating miR-375 levels and early alterations of islet integrity and functionality reflecting glucotoxicity in humans. However, this hypothesis has to be temporized as it is likely that only a small proportion (≈1%) of circulating miR-375 is originated from beta cells, at least in mice [33]. Extracellular miR-375 pool is very heterogeneous and various cells and tissues can release this miRNA. In human, miR-375 is also strongly expressed in GI-tract, adipose tissue, liver, adrenal, and brain [34]. Thus, altered circulating level of miR-375 at the beginning of T1D development could be also a general marker of metabolic alterations and/or inflammation associated with the disease, in addition to being representative of alterations of islet physiology. Indeed, most individuals affected by T1D exhibit multiple features associated with impaired B- and T-cell functions [35–37]. Presently, islet transplantation offers a potential cure for T1D [35]. Beside beta cell exhaustion and/or apoptosis, recurrent autoimmune damage creates a serious challenge to successful long-term islet transplantation [38]. The analysis of miRNAs changes (both in pancreas and blood) may help to find early biomarkers for beta cell damage.

miR-375 was also predicted to regulate pathways involved in viral infections in human islets. We have previously shown that circulating whole-blood cells from newly diagnosed patients with T1D, as well as autoantibody-positive first degree relatives, had increased expression of IFN-induced genes [39]. This fits well with the current knowledge of disease pathogenesis with the possible role played by viral infections [40]. Therefore, in addition to hyperglycemia and inflammation, expression of miR-375 might be also influenced by environmental factors such as viruses. In line with this hypothesis, miR-375 concentration in serum is modified in individuals infected with hepatitis B virus [41].

Taken together, our data demonstrated that miR-375 might be a potential biomarker at the onset of T1D that is also modulated in human islets exposed in vitro to glucose. It remains to determine whether this miRNA reflects only metabolic disturbances observed during T1D or also reflects chronic processes leading to inflammation of the pancreas and beta cell loss. Moreover, quantification of circulating miR-375 in prediabetic individuals is now necessary to validate the role of this new biomarker during the early phases of the disease.

## Supplementary Material

miRNA expressions in human islets quantified by using TaqMan Low Density arrays V3. Values are expressed as Ct.

## Figures and Tables

**Box 1:**
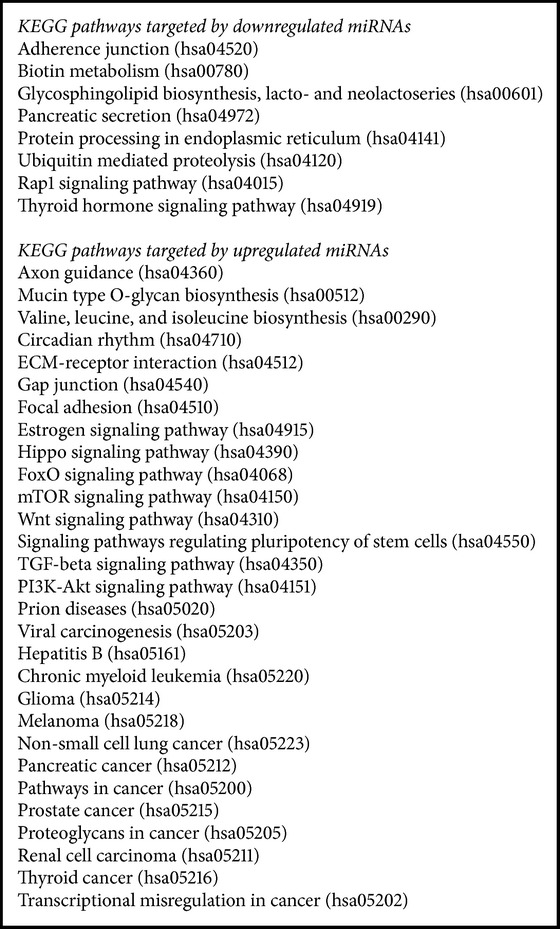
Significant KEGG pathways collectively targeted by miRNAs regulated by glucose in human islets, from DIANA miRPath v3.0 (http://snf-515788.vm.okeanos.grnet.gr/dianauniverse/index.php?r=mirpath).

**Table 1:** Clinical and metabolic characteristics of T1D children and of age-matched controls.

Parameters	Children with newly diagnosed T1D (*n* = 22)	Controls (*n* = 10)
Age (years)	9.81 ± 3.59	9.91 ± 2.34
Gender (M/F)	14/8	5/5
Glucose level during blood collection (mmol/L)	11.45 ± 3.48	4.0 ± 0.5^*∗*^
HbA1c (%)	11.82 ± 2.15	4.5 ± 0.5^*∗*^
BMI (kg/m^2^)	15.80 ± 2.49	ND
Number of patients with anti-GAD aAbs	*N* = 14	ND
Number of patients with anti-IA2 aAbs	*N* = 15	ND
Number of patients with anti-ZNT8 aAbs	*N* = 17	ND

^*∗*^ *p* < 0.05 T1D versus controls.

aAbs: autoantibodies.

**Table 2:** Characteristics of the islet donors.

Replicate ID	Age of the donners	Gender	BMI	% of beta cells in islets
P729	54	F	30.5	70
P733	23	M	26.0	76
P819	59	F	20.3	90

**Table 3:** Correlations between miR-375 concentration in sera and clinical and biological parameters of the T1D patients.

Variables	Pearson coefficient correlation	*p* values
Age	0.117	0.665
Gender	0.006	0.981
Glycaemia at discovery	0.212	0.430
HbA1c	0.236	0.378
BMI	0.187	0.487
Number of positive antibodies	0.130	0.629

BMI: body mass index.
