# Supplementary material for: miRNA-375 a Sensor of Glucotoxicity Is Altered in the Serum of Children with Newly Diagnosed Type 1 Diabetes
Source: J Diabetes Res. 2016 May 24;2016:1869082. doi: 10.1155/2016/1869082 (PMC4895032; doi:10.1155/2016/1869082)
Supplement: Supplementary file 1 — miRNA expressions in human islets quantified by using TaqMan Low Density arrays V3. Values are expressed as Ct. [file 1869082.f1.pdf]

Supplementary Table 1: miRNA expressions in human islets (n=3) quantified by using TaqMan Low Density arrays V3
Values are expressed as Ct. Ct levels are inversely proportional to the amount of target nucleic acid in the sample *(i.e .*; the lower the Ct level the greater the amount of target nucleic acid in the sample).

miRNA I.D	Mean expressions	SEM
hsa-miR-375-000564	11.4090424219759	0.182809537379463
hsa-miR-200c-002300	14.093484496006	0.234221559627277
hsa-miR-24-000402	14.1134897490604	0.231106915702722
hsa-miR-30b-000602	15.0949010659397	0.214068760388162
hsa-miR-30c-000419	15.4279378605923	0.394256492932443
hsa-miR-1274B-002884	15.4285383989337	0.400049818333967
hsa-miR-21-000397	15.4374777704743	0.372748988866631
hsa-miR-222-002276	15.4444014092248	0.217767863589828
hsa-miR-19b-000396	15.7687635818389	0.513220278550786
hsa-miR-29a-002112	16.0953071520909	0.178968231515176
hsa-miR-132-000457	16.0990403307267	0.184992717255149
hsa-miR-191-002299	16.1069318728026	0.188584596185825
hsa-miR-26a-000405	16.1125767927299	0.177743313349618
hsa-miR-200b-002251	16.1216044910452	0.184428357616407
hsa-miR-720-002895	16.4708311733682	0.316163771556956
hsa-let-7e-002406	16.4824487599673	0.211047871099023
hsa-miR-409-3p-002332	16.7748324541101	0.564883241030902
hsa-miR-125b-000449	16.7883268043984	0.392962416406514
hsa-miR-204-000508	16.7941683200965	0.383893193594959
hsa-miR-19a-000395	17.0965375676568	1.27116921031708
hsa-miR-146a-000468	17.1042475827262	0.672015180741739
hsa-miR-484-001821	17.1162101919164	0.158754789440184
hsa-miR-16-000391	17.1269269387294	0.15963744611319
hsa-miR-126-002228	17.1345214674815	0.502746733436366
hsa-miR-331-000545	17.148806407232	0.153186163272155
hsa-miR-30a-5p-000417	17.4497600544813	0.355031698288309
hsa-miR-200a-000502	17.464932964317	0.359791164175439
hsa-miR-127-000452	17.4652772900597	0.245587040186211
hsa-let-7b-002619	17.4975937975806	0.256589215367391
hsa-miR-342-3p-002260	17.8219757308135	0.370851152881512
hsa-miR-17-002308	18.1275018627528	0.130948315053603
hsa-miR-106a-002169	18.1317013083463	0.126772454023422
hsa-miR-145-002278	18.1368087278384	0.131407644987706
hsa-miR-574-3p-002349	18.1423665779698	0.12896413093285
hsa-miR-95-000433	18.1634788319214	0.136956556759084
mmu-miR-129-3p-001184	18.4453293273511	0.450756147416686
hsa-miR-141-000463	18.4532995882529	0.345910812870931
hsa-miR-148a-000470	18.4556713953297	0.344879375004883
hsa-miR-20a-000580	18.4649178286669	0.341727106450158
hsa-miR-197-000497	18.4735414037111	0.25867491313089
hsa-let-7a-000377	18.4820239479466	0.265224990315383
hsa-miR-376c-002122	18.4826212072075	0.345911650216952
hsa-miR-192-000491	18.4843354963091	0.350091825426071
hsa-miR-328-000543	18.520341892281	0.256924100053734
hsa-miR-194-000493	18.7855408929339	0.451822042173857
hsa-miR-539-001286	18.7868306701539	0.369797351574105
hsa-miR-30d-000420	18.7971852207831	0.574976745567401
hsa-miR-30e-3p-000422	18.8045470098177	0.0191827321762508
hsa-miR-320-002277	18.8075498668956	0.375735556507022
hsa-miR-382-000572	18.8098474734739	0.37904665676117

hsa-miR-30a-3p-000416	18.8220410183364	0.0165462579460382
hsa-miR-664-002897	18.8299808653899	0.564566622464326
dme-miR-7-000268	18.8481491231155	0.578978934127703
hsa-miR-221-000524	19.1265967105866	0.115563607446111
hsa-miR-26b-000407	19.1293273744793	0.114372142744331
hsa-miR-146b-001097	19.1332550330529	0.111002097925007
mmu-miR-374-5p-001319	19.1349065074427	0.110511046811392
hsa-miR-27b-000409	19.1371678890921	0.114672947667144
hsa-let-7g-002282	19.1403490266576	0.10907322751419
hsa-miR-193b-002367	19.1490271811442	0.111815714854385
hsa-miR-210-000512	19.4675115456953	0.326371923236268
hsa-miR-23b-000400	19.5030288601023	0.294599583047125
rno-miR-7*-001338	19.5151505281824	0.339117733483318
hsa-miR-181a-000480	19.5453455138915	0.351573035350457
hsa-miR-744-002324	19.8069638816045	0.367648579432308
hsa-miR-487b-001285	19.8120742733488	0.376803078844519
hsa-miR-151-3p-002254	19.8136708821324	0.0307385743274403
hsa-miR-1274A-002883	19.8206437617675	0.0377995189711924
hsa-miR-642-001592	19.8228365098135	0.367266515470931
hsa-miR-92a-000431	19.8330256297342	0.248323646065452
hsa-miR-27a-000408	19.8583675236368	0.245876480696219
hsa-miR-532-001518	20.1426328415336	0.0791857375462127
hsa-miR-660-001515	20.148841183087	0.076228797813496
hsa-miR-374-000563	20.1502214114813	0.0822827453750824
hsa-miR-365-001020	20.1510631056561	0.0989240773293898
hsa-miR-335-000546	20.1536234051327	0.089637245890432
hsa-miR-100-000437	20.154933111675	0.0629429540474335
hsa-miR-186-002285	20.1556099792453	0.0834534658322001
hsa-miR-411-001610	20.1568708706866	0.0838854315730358
hsa-miR-125a-5p-002198	20.1573129308247	0.0809357856335579
hsa-miR-28-3p-002446	20.1578218599755	0.0838485517857794
hsa-miR-22-000398	20.1586539620378	0.074095946511152
hsa-miR-532-3p-002355	20.1599698439452	0.0820821820640531
hsa-miR-429-001024	20.1605099099397	0.0780504127603588
hsa-miR-15b-000390	20.1610807113162	0.07997562873435
hsa-miR-103-000439	20.1634837035013	0.0897446609827177
hsa-miR-106b-000442	20.1654064238867	0.0813582080417763
hsa-miR-139-5p-002289	20.4728046306789	0.390959712142676
hsa-miR-432-001026	20.4740085624354	0.875464732893362
hsa-miR-652-002352	20.4842888711683	0.411615745894722
mmu-miR-140-001187	20.4990065971319	0.326887236112337
hsa-let-7d-002283	20.5051726875158	0.298225083351915
hsa-miR-301-000528	20.5130599520943	0.33258330656982
mmu-miR-134-001186	20.5218151010663	0.31307715749311
hsa-miR-31-002279	20.7895111506982	0.966470700271451
hsa-miR-199a-3p-002304	20.7957793207707	0.3956692131026
hsa-miR-216b-002326	20.8110570674509	0.356565634760327
hsa-miR-323-3p-002227	20.8172249161398	0.366679864914946
hsa-miR-410-001274	20.8244481007092	0.371819987433544
hsa-miR-370-002275	20.829075344931	0.359873538124385
hsa-miR-1275-002840	20.8293220982211	0.0672171615566211
hsa-miR-151-5P-002642	20.8447986367501	0.0572478385222907
hsa-miR-34a-000426	20.8479454950614	0.272255741862512
hsa-miR-376a-000565	20.8537921232473	0.388119534865556
hsa-miR-135a-000460	20.8615355070536	0.266427360234909
hsa-let-7f-000382	20.8709164330752	0.28908169500631
hsa-miR-129*-002298	21.1402459095193	0.64560185366345
hsa-miR-25-000403	21.1434579113167	0.0676519290551714
hsa-miR-29c-000587	21.1518876731175	0.612741388441448

hsa-miR-345-002186	21.1535968354312	0.0563561652785066
hsa-miR-455-3p-002244	21.1545349554228	0.0563814037438842
mmu-miR-495-001663	21.1571893460174	0.0497882895444903
hsa-miR-28-000411	21.1584042550473	0.0647266188691902
hsa-miR-296-000527	21.1604804214817	0.0622598087469461
mmu-miR-93-001090	21.1641437588525	0.0545822875079195
hsa-miR-598-001988	21.1656383274126	0.059840864679319
hsa-miR-184-000485	21.1722395479105	0.588458786447635
hsa-miR-324-5p-000539	21.1756148991838	0.0559327395737723
hsa-miR-149-002255	21.1788634467865	0.056417621845928
mmu-miR-379-001138	21.1806644524868	0.0620809239849145
hsa-miR-425-5p-001516	21.1871499793091	0.0507208234283464
hsa-miR-212-000515	21.1938434929201	0.0551130391606129
hsa-miR-223-002295	21.4923939096612	0.385561940176463
hsa-miR-455-001280	21.4964686395839	0.316305033409589
hsa-miR-324-3p-002161	21.5052441719353	0.321894634377141
hsa-miR-889-002202	21.5177396026014	0.321021522993072
hsa-let-7c-000379	21.5187812179964	0.308971217104676
hsa-miR-433-001028	21.8220859971244	0.355412920282791
hsa-miR-143-002249	21.826416343723	0.367255543648316
hsa-miR-218-000521	21.8271817358663	0.653158081399374
hsa-miR-655-001612	21.8341220908569	0.359742068386875
hsa-miR-494-002365	21.8343903215668	0.360732998157617
hsa-miR-7-2*-002314	21.8412729042626	0.577275939531352
hsa-miR-183*-002270	21.8418466183342	0.56474494934696
hsa-miR-15a-000389	21.8760034099664	0.295755348540836
hsa-miR-339-5p-002257	22.1567105142001	0.0593633184251075
hsa-miR-874-002268	22.1642855772968	0.0394031477112529
hsa-miR-130b-000456	22.1647765920985	0.0251994920697329
hsa-miR-183-002269	22.1677995711843	0.052106489447032
hsa-miR-152-000475	22.1690147421133	0.0390010927037157
hsa-miR-485-3p-001277	22.1695023884322	0.0454927396755524
hsa-miR-339-3p-002184	22.1705083199227	0.0416750076522881
hsa-miR-29b-000413	22.1714366728698	0.585948358386286
hsa-miR-214-002306	22.1760226566728	0.0413183075290311
hsa-miR-203-000507	22.1773107271859	0.0421856985457947
hsa-miR-136*-002100	22.18175515683	0.30808422029096
hsa-miR-133a-002246	22.4978620652156	0.378346212990015
hsa-miR-340-002258	22.5028198816303	0.316671062348874
hsa-miR-10a-000387	22.508254528335	0.317088176220471
hsa-miR-135b-002261	22.514186578841	0.319041283094307
hsa-miR-485-5p-001036	22.5215244180707	0.340107954760916
mmu-miR-491-001630	22.5215437246323	0.348087540271882
hsa-miR-766-001986	22.5229852185879	0.397454252868143
hsa-miR-129-000590	22.5326825430302	0.396073926811334
hsa-miR-150-000473	22.5349403774384	0.313912693568888
hsa-miR-193a-5p-002281	22.5420603810988	0.345928083303635
hsa-miR-1260-002896	22.5469025518061	0.39760664406623
hsa-miR-885-5p-002296	22.5493037277487	0.922330474986492
hsa-miR-130a-000454	22.8320590885957	0.342635759284835
hsa-miR-195-000494	22.8352239401163	0.34777725090094
hsa-miR-668-001992	22.8403395200663	0.531919265103061
hsa-miR-182-002334	22.8407429558257	0.363371847835187
hsa-miR-20b-001014	22.8414823183821	0.356507781131756
hsa-miR-708-002341	22.8423612688369	0.369735234508271
hsa-miR-128a-002216	22.8489245524679	0.353856108231434
hsa-miR-590-5p-001984	22.8490169564101	0.651645709168613
hsa-miR-361-000554	22.8509027613104	0.366932860319354
hsa-miR-148b-000471	22.8608666659115	0.307777757414557

hsa-miR-1180-002847	22.8623594194136	0.0994018431832439
hsa-miR-1233-002768	22.8650095170226	0.506262335272709
hsa-miR-98-000577	22.8851432498905	0.844617401068388
hsa-miR-15b*-002173	22.892455660711	0.0803087146497523
hsa-miR-454-002323	23.158909752713	0.595497896840699
hsa-miR-217-002337	23.1638628242705	0.601284002796708
rno-miR-29c*-001818	23.1660692382459	0.289200465805054
hsa-miR-99b*-002196	23.1734981481353	1.42181852457395
hsa-miR-22*-002301	23.1775812173102	0.308125098112742
hsa-miR-200a*-001011	23.1783180101893	0.28015839656959
hsa-miR-155-002623	23.1808287041578	0.0259622697063724
hsa-miR-185-002271	23.1868133621666	0.0219925841781481
hsa-miR-27b*-002174	23.195834306372	0.282761972376776
hsa-miR-126*-000451	23.2015315249484	0.873958925427038
hsa-miR-625*-002432	23.2046236762441	0.706919211545271
hsa-miR-335*-002185	23.2054391236521	0.276071166699022
hsa-miR-320B-002844	23.4951556270779	0.811118364971755
hsa-miR-551b-001535	23.4999716204682	0.313058505211061
hsa-miR-224-002099	23.5117329066639	0.320765856395697
hsa-miR-489-002358	23.5143812471561	0.335664751067865
hsa-miR-340*-002259	23.5158986580343	0.243702245466632
hsa-miR-27a*-002445	23.5179634270783	0.251648611670133
hsa-miR-590-3P-002677	23.5255698013453	0.237095460568039
hsa-miR-101-002253	23.5267208735724	0.309679909992124
hsa-miR-1271-002779	23.5290904170373	0.384476666228597
hsa-miR-10b-002218	23.537846021154	0.319206476814607
hsa-miR-886-3p-002194	23.5381299634128	0.371776439572636
hsa-miR-423-5p-002340	23.539618046444	0.364378393448899
hsa-miR-654-001611	23.5451070652327	0.313533982562251
mmu-miR-137-001129	23.8256979586283	0.321047558126646
hsa-miR-483-5p-002338	23.8436203866853	0.361896089172278
hsa-miR-142-3p-000464	23.8621685329203	0.64588188734059
hsa-miR-362-001273	23.8640285992258	0.37383053864676
hsa-miR-93*-002139	23.8669304324743	0.577442824365726
hsa-miR-222*-002097	23.8697132285042	0.115368390627265
hsa-miR-501-001047	23.874412691844	0.342624218759745
hsa-miR-34a*-002316	23.8863251014363	0.567800234622023
hsa-miR-886-5p-002193	23.8867035189974	0.719025594583823
hsa-miR-106b*-002380	24.2043562687635	0.28816028564568
hsa-miR-1290-002863	24.2166564233315	0.460379601435911
hsa-miR-31*-002113	24.4966070270904	0.789751188221013
hsa-miR-9-000583	24.5002659133822	0.635589884196991
hsa-miR-140-3p-002234	24.5268007232618	0.320990369888649
hsa-miR-592-001546	24.5270410276174	0.426070290682527
hsa-miR-337-5p-002156	24.5311372610637	0.31599095137021
hsa-miR-216a-002220	24.5358927732951	0.304315427392452
hsa-miR-181a-2*-002317	24.5468140073005	0.222352256396551
hsa-miR-130b*-002114	24.5567093419755	0.415174987011857
hsa-miR-500-002428	24.5635591737225	0.287533774443017
hsa-miR-18a-002422	24.8531217646854	0.303587935245422
hsa-miR-190b-002263	24.8604092716011	0.131664853584986
hsa-miR-628-5p-002433	24.8752905684645	0.373561796450224
hsa-miR-543-002376	24.8764911089103	0.129815612482662
hsa-miR-769-5p-001998	24.8780631749615	0.126734721591182
hsa-miR-192*-002272	24.8813588300841	0.133816827850943
hsa-miR-656-001510	24.8845398350725	0.605192447382104
hsa-miR-409-5p-002331	24.8880755609372	0.375435960107633
hsa-miR-502-3p-002083	25.1899701677052	0.0508459719239248
hsa-miR-383-000573	25.1957398033033	0.5807663496547

hsa-miR-758-001990	25.1970146656219	0.0525542481161999
hsa-miR-671-3p-002322	25.2028081691425	0.0513957104947115
hsa-miR-107-000443	25.2114896490784	0.0738270063011371
hsa-miR-493-002364	25.2146318738192	0.0568344802474741
hsa-miR-1179-002776	25.2172527569999	0.278376528899133
hsa-miR-26b*-002444	25.2180421928068	0.283380047061739
hsa-miR-342-5p-002147	25.2200746109994	0.633967740214679
hsa-miR-577-002675	25.2202694484962	0.272478071529951
hsa-miR-30d*-002305	25.2218934644959	0.306502882221056
hsa-miR-26a-1*-002443	25.2280046227179	0.747817071166143
hsa-miR-628-3p-002434	25.2306657534156	1.23805097224449
hsa-miR-200b*-002274	25.4685222421818	3.21755065606683
hsa-miR-338-3p-002252	25.5226544945415	0.315525030680193
hsa-miR-362-3p-002117	25.5413394581903	0.315814517797675
mmu-miR-96-000186	25.544252703943	0.297311642909914
hsa-miR-337-3p-002157	25.5521711141658	0.434686380056238
hsa-miR-629-001562	25.5532421049071	0.413254145522143
hsa-miR-770-5p-002002	25.5536833224393	0.907677258006187
hsa-miR-1201-002781	25.5577506193428	0.196350054216527
hsa-miR-625-002431	25.5633044742756	0.407045243333541
hsa-miR-523-002386	25.5695453879186	0.414926840488736
hsa-miR-301b-002392	25.8668327567851	0.283511800062776
hsa-miR-138-002284	25.8673034795056	0.274885957903324
hsa-miR-378-002243	25.8726866455362	0.149552990858376
hsa-miR-425*-002302	25.8812587572809	0.585045431883207
hsa-miR-29a*-002447	25.8849696260884	0.60022864124257
hsa-miR-154-000477	25.9079850405786	0.369538956343245
mmu-miR-153-001191	26.2079802782619	0.524778584103946
hsa-miR-125a-3p-002199	26.2147191945572	0.0739075171240879
hsa-miR-744*-002325	26.215047758475	0.280081751570348
hsa-miR-148b*-002160	26.2159471704175	0.275623287474995
hsa-miR-452-002329	26.218405753228	0.0827062754882851
hsa-miR-10b*-002315	26.2189204162048	0.27483317958235
hsa-miR-15a*-002419	26.2204322971522	0.29251030249047
hsa-miR-411*-002238	26.2246342417578	0.271414143713501
hsa-miR-20a*-002437	26.2270954533539	0.292897636267212
hsa-miR-487a-001279	26.238995169716	0.0800170247252919
hsa-miR-194*-002379	26.2397657512174	0.29824540157288
mmu-miR-187-001193	26.5468181598508	0.27099135974762
hsa-miR-505*-002087	26.5542837401737	0.440488898579504
hsa-miR-24-2*-002441	26.5567098483002	0.174339084607129
hsa-miR-486-001278	26.5654104484559	0.441906606566778
hsa-miR-29b-2*-002166	26.5763957040364	0.453067937507089
hsa-miR-551b*-002346	26.5766682944917	0.432451885508622
hsa-miR-338-5P-002658	26.8845350228574	0.164743296148686
hsa-miR-18a*-002423	26.8903285274931	0.447163747344664
hsa-miR-33a*-002136	26.890755727338	0.177669787165707
hsa-miR-597-001551	26.891867498501	0.635597843572423
hsa-miR-34b-002102	26.8924725133612	0.45800273344477
hsa-miR-454*-001996	26.8956530782042	0.586728619727933
hsa-miR-1254-002818	26.8959638314307	0.173016892998893
hsa-miR-1285-002822	26.9008688132568	0.187727197862123
hsa-miR-193b*-002366	26.9270000463875	0.586428730708478
hsa-miR-16-1*-002420	27.1942306958985	0.517003517796375
hsa-miR-191*-002678	27.2100948098121	0.278674654037944
hsa-miR-579-002398	27.2179322231304	0.108313190768649
hsa-miR-19b-1*-002425	27.2218599199635	0.303176435494796
hsa-miR-145*-002149	27.2224705621218	0.295825632615731
hsa-miR-891a-002191	27.2235682745656	0.106682863870426

hsa-miR-329-001101	27.2289325872455	0.105802633951673
hsa-miR-505-002089	27.230336481435	0.114286468292848
hsa-miR-654-3p-002239	27.2313915123828	0.105519786033896
hsa-miR-381-000571	27.2332241959513	0.102223824728624
hsa-miR-213-000516	27.2373523010076	0.296319379230575
hsa-miR-380-5p-000570	27.2471256407948	0.286970759010159
hsa-miR-942-002187	27.552902790608	0.451468995090841
hsa-miR-141*-002145	27.5614762393776	0.160689484650308
hsa-miR-497-001043	27.5635577804082	0.611086646349702
hsa-miR-214*-002293	27.5695247328002	0.170753999539957
hsa-miR-1270-002807	27.5713757599633	0.449919314205901
hsa-miR-1-002222	27.5793690451588	0.256665417685925
hsa-miR-500-001046	27.586327318307	0.462760127739945
hsa-miR-377-000566	27.5964327718087	0.248628121708723
hsa-miR-424*-002309	27.6223024610923	0.867792615673548
hsa-miR-9*-002231	27.8860096382716	0.430048151430275
hsa-miR-17*-002421	27.8953040674828	0.447727240429677
hsa-miR-32-002109	27.9042794357625	0.657470320029399
hsa-miR-424-000604	27.9144294996028	0.387076554933671
hsa-miR-1226*-002758	27.9150286134639	0.595206280212977
hsa-miR-541-002201	27.9167607172861	0.381134683766797
hsa-miR-589-001543	27.9170751809285	0.183866994906644
hsa-miR-99a*-002141	27.9273535496924	0.189084322362001
hsa-miR-380-3p-000569	27.9426730071321	0.915513121996711
hsa-miR-618-001593	28.2285590445057	0.135894663787753
hsa-miR-876-3p-002225	28.2306060503668	0.132993553683898
hsa-miR-154*-000478	28.2390932962017	0.295504336609007
mmu-miR-499-001352	28.2428133401724	0.555194277088917
hsa-miR-330-5p-002230	28.2477499650532	0.132576124139899
hsa-miR-542-3p-001284	28.2483765211166	0.696350367083218
hsa-miR-135b*-002159	28.2484552530406	0.309392690641575
hsa-miR-511-001111	28.5457973919167	0.2491825126981
hsa-miR-146b-3p-002361	28.5636394765366	0.321430492940869
hsa-miR-190-000489	28.57893284157	0.326952114030758
hsa-miR-125b-1*-002378	28.5798380724786	0.132714444376395
hsa-miR-873-002356	28.5812875467831	0.483461088331925
hsa-miR-363-001271	28.5838248997542	0.329633730910833
hsa-miR-369-3p-000557	28.5853570854557	0.330174863768209
hsa-miR-876-5p-002205	28.5886211567799	0.481917001355806
hsa-miR-1300-002902	28.5886492971276	0.44419301700959
hsa-miR-503-001048	28.5980314742355	0.482849499619387
hsa-miR-132*-002132	28.6022292296972	0.479454172541422
hsa-miR-453-002318	28.8775934959113	0.199680502304858
hsa-miR-520D-3P-002743	28.8843977522194	1.41705113943455
hsa-miR-1197-002810	28.8945799274917	0.619581444871978
hsa-miR-616-002414	28.9158773654739	0.444420924481143
hsa-miR-1303-002792	28.9162021122352	0.618738114220077
hsa-miR-376a*-002127	28.9223181282617	0.617937007433571
hsa-miR-512-3p-001823	28.9279160922019	0.226155370994928
hsa-miR-369-5p-001021	28.9309216472652	0.413273728486994
hsa-miR-545-002267	29.2309447106428	0.984697823786375
mmu-miR-496-001953	29.2484719891592	0.153482485165239
hsa-miR-576-3p-002351	29.2531481450111	0.159744477943335
hsa-miR-802-002004	29.2569042398566	0.297015118363453
hsa-miR-147b-002262	29.2606050014682	0.158402834228673
hsa-miR-127-5p-002229	29.2611461547427	0.152810405043257
mmu-miR-451-001141	29.2623232344012	0.737407128159734
hsa-miR-616-001589	29.2654742972546	0.304070895159879
hsa-miR-450b-5p-002207	29.2713651316618	0.720576042316267

hsa-miR-1291-002838	29.2818079321318	0.875833823223302
hsa-miR-488-002357	29.5497319652264	0.535100263511713
hsa-miR-374a*-002125	29.5598816245156	0.603911897552144
hsa-miR-517a-002402	29.5672368095283	0.546552860882747
hsa-let-7i*-002172	29.5755290172632	0.118509985208285
hsa-miR-544-002265	29.5822899979736	0.337943309038341
hsa-miR-1248-002870	29.5929820737845	0.955563079535068
hsa-miR-29b-1*-002165	29.6007210717693	0.489420452280929
hsa-miR-589-002409	29.6008301575357	0.508776435820132
hsa-miR-206-000510	29.8901747510474	1.14643834750509
hsa-miR-550-001544	29.9103782474548	0.611085648182251
hsa-miR-21*-002438	29.9185129448406	1.01987663211062
hsa-miR-938-002181	29.9232943343771	0.224008578555611
hsa-miR-888-002212	29.9263577865252	0.193081621428335
hsa-miR-26a-2*-002115	29.9313359502593	0.626518111165838
hsa-miR-1255B-002801	29.9327181266186	0.232691937486548
hsa-miR-629-002436	29.9401431535052	0.477918842632935
hsa-miR-221*-002096	29.9474428992803	0.63066993931932
hsa-miR-122-002245	29.9489009354728	0.425276545189965
hsa-miR-193a-3p-002250	29.9492255277921	0.647870293282961
hsa-miR-219-000522	29.9732671926094	0.483090471692138
hsa-miR-92a-1*-002137	30.2485533350249	0.306933873629655
hsa-let-7f-2*-002418	30.2522271706802	0.309307129764978
hsa-miR-542-5p-002240	30.293388394879	0.733711033983622
hsa-miR-550-002410	30.5959621922311	0.578963088143269
hsa-miR-450a-002303	30.6123313417048	0.521972303838087
hsa-miR-34c-000428	30.6314403244754	0.337743588536212
hsa-miR-372-000560	30.8824990462087	0.703788743430769
hsa-miR-519a-002415	30.9082276761173	0.771037259048753
hsa-miR-944-002189	30.9361384397778	0.611077275281035
hsa-miR-422a-002297	30.9428011974893	0.419888714931589
hsa-miR-1269-002789	30.9556161069313	0.630935563218197
hsa-let-7e*-002407	30.9568751331653	0.643582974595983
hsa-miR-378-000567	30.9574732938174	0.648936425208819
hsa-miR-377*-002128	30.9588776853674	0.243666349244201
hsa-miR-548b-5p-002408	30.9630841963681	0.51375959367261
hsa-miR-875-5p-002203	31.2642473316916	0.314462414449595
hsa-miR-653-002292	31.2659421063981	0.197653222046665
hsa-miR-10a*-002288	31.2722024885472	0.304318691242213
hsa-miR-572-001614	31.2748084128571	0.299843996595539
hsa-miR-331-5p-002233	31.281278583351	0.203301308319172
hsa-miR-1276-002843	31.2897448936428	0.281695382819698
hsa-miR-601-001558	31.5688321908737	0.678165954181554
hsa-miR-296-3p-002101	31.5881440972634	0.735787714223993
hsa-miR-144*-002148	31.6272668155588	0.502529907972765
hsa-miR-548c-5p-002429	31.6435363098417	0.832760955724481
hsa-miR-517c-001153	31.9276607169614	0.1695038013774
hsa-miR-570-002347	31.9285710307898	0.137702626456949
hsa-miR-374b*-002391	31.9354150720021	0.660043280175497
hsa-miR-519d-002403	31.9374556493588	0.675738452961195
hsa-miR-142-5p-002248	31.9784477984023	0.511450599291099
hsa-miR-624-001557	32.2230619578564	1.71712588950822
hsa-miR-23a*-002439	32.2647549796874	0.711348040751075
hsa-miR-449-001030	32.2671004296342	0.4786596908938
hsa-miR-548d-5p-002237	32.292205361569	0.239635157982789
hsa-miR-519b-3p-002384	32.5994168909054	0.670113876911554
hsa-miR-1244-002791	32.6072386244753	0.590757151067653
hsa-miR-449b-001608	32.6227298689224	0.376368672933425
hsa-miR-627-001560	32.6231359402235	0.349541376081705

hsa-miR-19a*-002424	32.9701182424223	0.660403293011517
hsa-miR-147-000469	32.9887769976338	0.556186342169823
hsa-miR-520c-3p-002400	33.2752897333262	0.402744123935107
hsa-miR-651-001604	33.3028951372187	0.255848033563836
hsa-miR-100*-002142	33.5962102839617	1.04513563593823
hsa-miR-582-3p-002399	36.381970804522	0.905050776000067
**other small RNAs**	**Mean expressions**	**SEM**
U6 snRNA-001973	12.6437093879987	0.32846467025933
RNU48-001006	13.4416835520095	0.192852024367005
RNU44-001094	16.1147346053091	0.377671789580817
